# Efficacy of Integrated Social Cognitive Remediation vs. Neurocognitive Remediation in Improving Functional Outcome in Schizophrenia: Concept and Design of a Multicenter, Single-Blind RCT (The ISST Study)

**DOI:** 10.3389/fpsyt.2022.909370

**Published:** 2022-06-21

**Authors:** Wolfgang Wölwer, Nicole Frommann, Agnes Lowe, Daniel Kamp, Karolin Weide, Andreas Bechdolf, Anke Brockhaus-Dumke, Rene Hurlemann, Ana Muthesius, Stefan Klingberg, Martin Hellmich, Sabine Schmied, Andreas Meyer-Lindenberg, For the ISST study group, W. Wölwer

**Affiliations:** ^1^Department of Psychiatry and Psychotherapy, Medical Faculty, University of Düsseldorf, Düsseldorf, Germany; ^2^Department of Psychiatry, Psychotherapy and Psychosomatic Medicine Incorporating FRITZ and Soulspace, Vivantes Klinikum Am Urban, Berlin, Germany; ^3^Department of Psychiatry, Psychotherapy and Psychosomatic Medicine, Rheinhessen-Fachklinik Alzey, Alzey, Germany; ^4^Department of Psychiatry and Psychotherapy, LVR-Klinik Bonn, Bonn, Germany; ^5^Department of Psychiatry and Psychotherapy, University of Bonn, Bonn, Germany; ^6^Department of Psychiatry and Psychotherapy, University of Oldenburg, Oldenburg, Germany; ^7^Department of Psychiatry and Psychotherapy, University of Cologne, Cologne, Germany; ^8^Department of Psychiatry and Psychotherapy, University of Tübingen, Tübingen, Germany; ^9^Institute of Medical Statistics and Computational Biology, Faculty of Medicine and University Hospital of Cologne, University of Cologne, Cologne, Germany; ^10^Clinical Trials Center, University of Cologne, Cologne, Germany; ^11^Department of Psychiatry and Psychotherapy, Central Institute for Mental Health, Medical Faculty Mannheim, Heidelberg University, Mannheim, Germany

**Keywords:** schizophrenia, cognitive remediation, social cognition, social skills, functional outcome, recovery

## Abstract

**Background:**

Although clinically effective treatment is available for schizophrenia, recovery often is still hampered by persistent poor psychosocial functioning, which in turn is limited by impairments in neurocognition, social cognition, and social behavioral skills. Although cognitive remediation has shown general efficacy in improving cognition and social functioning, effects still need to be improved and replicated in appropriately powered, methodologically rigorous randomized controlled trials (RCTs). Existing evidence indicates that effects can most likely be optimized by combining treatment approaches to simultaneously address both social cognitive and social behavioral processes.

**Objectives:**

To assess whether Integrated Social Cognitive and Behavioral Skill Therapy (ISST) is more efficacious in improving functional outcome in schizophrenia than the active control treatment Neurocognitive Remediation Therapy (NCRT).

**Methods:**

The present study is a multicenter, prospective, rater-blinded, two-arm RCT being conducted at six academic study sites in Germany. A sample of 180 at least partly remitted patients with schizophrenia are randomly assigned to either ISST or NCRT. ISST is a compensatory, strategy-based program that targets social cognitive processes and social behavioral skills. NCRT comprises mainly drill and practice-oriented neurocognitive training. Both treatments consist of 18 sessions over 6 months, and participants are subsequently followed up for another 6 months. The primary outcome is all-cause discontinuation over the 12-month study period; psychosocial functioning, quality of life, neurocognitive and social cognitive performance, and clinical symptoms are assessed as secondary outcomes at baseline before randomization (V1), at the end of the six-month treatment period (V6), and at the six-month follow-up (V12).

**Discussion:**

This RCT is part of the German Enhancing Schizophrenia Prevention and Recovery through Innovative Treatments (ESPRIT) research network, which aims at using innovative treatments to enhance prevention and recovery in patients with schizophrenia. Because this study is one of the largest and methodologically most rigorous RCTs on the efficacy of cognitive remediation approaches in schizophrenia, it will not only help to identify the optimal treatment options for improving psychosocial functioning and thus recovery in patients but also allow conclusions to be drawn about factors influencing and mediating the effects of cognitive remediation in these patients.

**Trial Registration:**

ClinicalTrials.gov NCT 02678858, German Study Register DRKS 00010033

## Introduction

### Background

Schizophrenia is a severe mental disorder that places significant burden on affected individuals, their families, and the community. Beyond the clinical symptoms, individuals with schizophrenia experience severe social disabilities that profoundly impact their quality of life and limit recovery (1, 2). Impairments in social functioning affect a wide range of domains comprising, e.g., interpersonal behavior in the community or laboratory, independent living, and social problem solving. Even though available treatment strategies are effective in reducing clinical symptoms, social functioning often remains significantly impaired. In particular, antipsychotic medication has only marginal impacts on social functioning and subjective quality of life (3, 4). On the other hand, patients self-rate social functioning as their area of greatest unmet need (2, 5). Thus, there is an urgent need for treatment optimization. As modern treatment concepts more and more aim at full recovery rather than mere reduction of clinical symptoms multi-component treatment packages are being developed (6–8). Treatments that directly target key determinants of functional outcome, such as cognitive functioning, social behavioral skills, and negative symptoms, seem to be the most promising components of such approaches.

Besides negative symptoms, basic cognitive functioning (e.g., attention, memory, and executive functions, together often referred to as *neurocognition* or *nonsocial cognition*), and, to an even greater extent, social cognitive processes (defined as the mental operations underlying social interaction, such as social perception, affect recognition, and theory of mind [ToM]) are the most potent determinants of functional outcome (9). In particular, social cognitive impairments are closely associated with impaired social functioning in schizophrenia (10–12). Within social cognition, the domains that are known to be most impaired, i.e., facial and prosodic affect recognition, social perception, and ToM (13), are most closely associated with functional outcome (14). Furthermore, cognitive impairments are associated not only with worse social functioning but also with worse therapy adherence (15) and less service engagement (16). Cognitive functioning and therapy adherence both act as mediators or moderators of treatment effects; strictly speaking, they can even be considered as preconditions of any treatment effects because patients can only benefit from potentially effective treatment if they actually use it and show good adherence. Thus, poor service engagement and treatment adherence are highly relevant problems in the treatment and care of patients with schizophrenia, in particular given that up to two thirds of patients with schizophrenia do not participate in potentially effective therapies or discontinue them early (17), a finding that is especially true after the first episode of schizophrenia.

In the past two decades, a number of cognitive remediation programs that systematically target impairments in neurocognition and/or social cognition have been developed to improve cognitive functioning. By improving cognition, these programs ultimately strive to achieve lasting benefits in community functioning. Several meta-analyses quite consistently showed that cognitive remediation successfully improves the targeted cognitive domains of basic or social cognition. Effect sizes were reported to be moderate (d = 0.45) in meta-analyses of studies on predominately neurocognitive remediation (18), but lower (d = 0.29) in a recent meta-analysis that aggregated various kinds of cognitive remediation programs, including metacognitive training (19). Meta-analyses focusing on social cognitive remediation often reported more pronounced effects (up to d = 0.42–1.35, depending on the social cognitive domain and type of training), although they included fewer studies (20–22). In particular, Training of Affect Recognition (TAR)—a precursor of the treatment used in the present study—has proven its efficacy in several studies with regard to recognition of facial affect (23, 24), recognition of prosodic affect and ToM (25, 26), and improvement in subjective quality of life (27).

Beyond cognitive processes, Brekke et al. (28) describe social competence or social skills, i.e., the skills that people need to interact and communicate with others, as additional significant predictors of functional outcome in schizophrenia. Whereas cognitive remediation programs aim to improve functional outcome by improving cognitive processes, conventional social skills training primarily focuses on behavioral rehearsal, positive reinforcement, and corrective feedback about socially compatible behavior; however, typically it does not explicitly address underlying cognitive processes. Also, such social skills training has shown efficacy in proximal measures of behavioral skills: A meta-analysis revealed a large effect size for content mastery exams (d = 1.20) and a moderate effect size for performance-based measures of social and daily living skills (d = 0.52) (29).

With regard to more distal outcomes of social functioning, both cognitive remediation and social skills training have yielded statistically significant but clinically as yet unsatisfactory effects. With respect to improvements in social and daily living skills or community function, meta-analyses revealed effect sizes of d = 0.52 for social skills training (29), d = 0.42 for studies primarily targeting neurocognition (18), and d = 0.78 to 0.82 for social cognitive remediation (20, 22). However, a recent meta-analysis failed to find significant effects of social cognitive remediation on psychosocial functioning (30). The potential superiority of social cognitive over neurocognitive remediation in improving functional outcome, as shown by most of these meta-analyses, was confirmed in a head-to-head comparison in an own study of TAR: Compared with a neurocognitive remediation program, TAR achieved larger improvements in functional outcome with a between-treatment effect size of d = 0.58 (25); however, the sample was very small (*n* = 38), and these findings need to be replicated.

Moderator analyses of recent reviews or meta-analyses (18, 19, 31) suggest that the effects of cognitive remediation on functional outcome may be significantly enhanced by combining it with social behavioral skills training or other rehabilitative measures and by practicing these skills in the community. However, so far only a few prospective studies have systematically investigated these assumptions. Whereas Horan et al. (32) failed to enhance generalization to functional improvements through in vivo community-based cognitive remediation, Bowie et al. (33) observed a beneficial effect on functional behavior by combining cognitive remediation and functional skills training: Compared with neurocognitive remediation followed by treatment as usual, a program consisting of neurocognitive remediation followed by functional skills training led to a larger improvement in observer-rated community activities and work skills—but not interpersonal behavior-−12 weeks after the end of treatment.

### Objectives

First evidence indicates that combined social cognitive remediation and social behavioral skill training is the most promising approach to obtain clinical improvements in social functioning. However, this evidence needs corroborating and extending. In particular, consecutive application of both interventions (as used by 33) may miss potential additional benefits from parallel application. Moreover, social cognitive remediation may be more effective in schizophrenia than neurocognitive remediation because social cognition is more closely related to functional outcome than neurocognition is (14) and social cognitive remediation has shown larger effects on social functioning than neurocognitive remediation has (18, 20). Thus, the major objective of this trial is to investigate whether integrated social cognitive remediation and social behavioral skills therapy is more efficacious than neurocognitive remediation (as an active control treatment) in improving functional outcome and therapy adherence (i.e. the continuation or discontinuation of treatment). Moreover, the trial will investigate the durability of functional improvements and the treatment effects on quality of life and neurocognitive, social cognitive, and social behavioral performance.

## Methods

### Study Design

**Figure 1:**
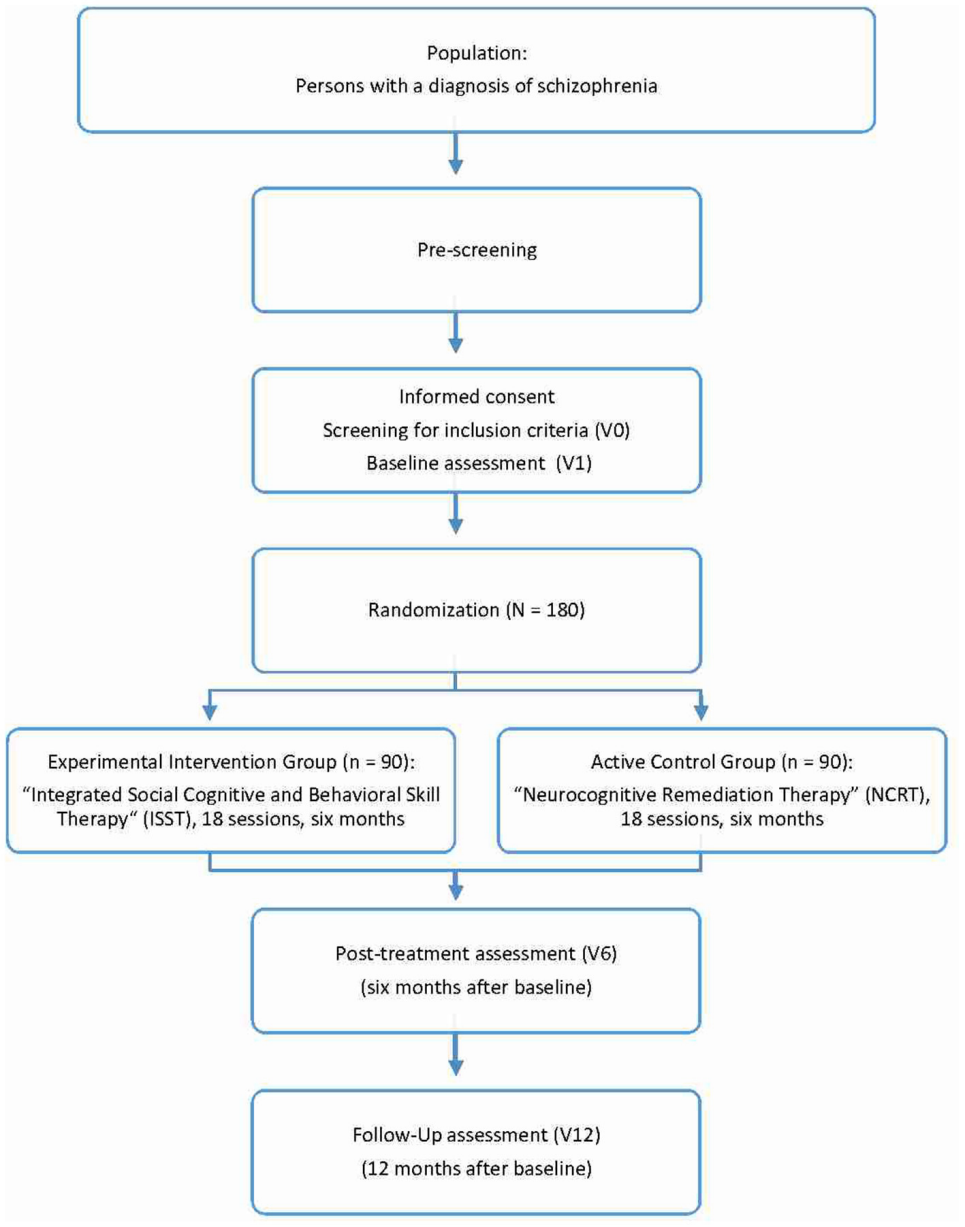
Overview of the trial.

This is a multicenter, prospective, single-blind, parallel-group, randomized clinical trial (RCT) comparing the experimental intervention Integrated Social Cognitive and Behavioral Skill Therapy (ISST) and the active control intervention Neurocognitive Remediation Therapy (NCRT) with respect to their efficacy in improving treatment adherence and functional outcome in schizophrenia. Outcomes are assessed at baseline before randomization (V1), at the end of the six-month treatment period (V6), and at the 6-month follow-up (V12; Figure 1).

The study is being conducted at six study sites in Germany, i.e., the University Departments of Psychiatry and Psychotherapy in Düsseldorf (coordinating site; Coordinating Investigator [CI], W. Wölwer), Bonn (Principal Investigator [PI], R. Hurlemann), Cologne (PI, A. Muthesius), and Tübingen (PI, S. Klingberg) and the specialized academic hospitals of psychiatry in Alzey (PI: A. Brockhaus-Dumke) and Berlin (PI: A. Bechdolf). The study is part of the national “Enhancing Schizophrenia Prevention and Recovery through Innovative Treatments” (ESPRIT) research network (coordinator: A. Meyer-Lindenberg, Mannheim), which is funded by the German Federal Ministry of Education and Research (Bundesministerium für Bildung und Forschung [BMBF]). The study has received approval from the local ethics committees and is being carried out in accordance with good clinical practice principles and the latest version of the Declaration of Helsinki. Before recruitment started, the study was registered at ClinicalTrials.gov (NCT 02678858) and in the German Clinical Trials Register (DRKS 00010033).

### Participant Selection and Inclusion and Exclusion Criteria

Patients are recruited from the in- and outpatient facilities of the participating institutions, where all patients with a diagnosis of schizophrenia are pre-screened for eligibility. Eligible patients are informed orally and in writing about the study. If they show interest in participating, written consent is obtained before inclusion and exclusion criteria are formally examined (assessment V0). Patients with a legal guardian are only included if both the patient and the legal guardian give their informed consent.

In addition to providing informed consent, to be included in an ESPRIT network study patients have to meet the diagnostic criteria for schizophrenia (Diagnostic and Statistical Manual of Mental Disorders, Fourth Edition, Text Revision, DSM-IV-TR: 295.10–30, 295.90) in a standardized diagnostic interview (Mini International Neuropsychiatric Interview MINI version 6.0). Other inclusion criteria are fluency in German, age between 18 and 65 years, a verbal intelligence quotient above 80, stable medication for at least 2 weeks with no more than two antipsychotics, and a maximum Positive and Negative Syndrome Scale (PANSS) score of 75 at baseline. Exclusion criteria are an impaired ability to consent, a positive drug screening (apart from a positive test for cannabis, amphetamines, and benzodiazepines) in the recruitment process, severe risk of suicide at the baseline visit, other severe axis I psychiatric diagnoses, and severe neurological or somatic comorbidities. An additional exclusion criterion for the present study is receiving psychotherapy (or having received it within the last 2 months) that is comparable to the experimental study treatment, e.g., social skills training or social cognitive remediation.

### Allocation of Participants to Treatment Conditions

All patients who fulfill the inclusion criteria and give written informed consent are randomized to one of the two treatment conditions in a 1:1 ratio. The allocation sequence is automatically generated by the internet service ALEA by applying a permuted block design with random blocks of varying length stratified by study center. The initial setup was independently programmed by the Institute of Medical Statistics and Computational Biology (IMSB) at the University of Cologne (Head: M. Hellmich). The randomization process for each patient is initiated by the therapist, who also is the only person to be informed about group allocation. The therapist then shares this information with the patient, but never with the raters. Patients are explicitly asked not to disclose their treatment condition during assessment visits with the raters. Raters are not allowed to conduct treatment sessions, and therapists are not involved in assessing outcomes. Furthermore, the raters must complete a so-called blindness protocol after each visit. Thus, this study strictly adheres to a single-blind design by completely separating treatment and assessment.

### Treatment Conditions

The experimental intervention ISST targets expressive and interactional behavior skills and the social cognitive domains known to be most impaired in schizophrenia (i.e., facial and prosodic affect recognition, social perception, and ToM) (13) and most closely associated with functional outcome (14). ISST is primarily based on the social cognitive remediation program TAR, which was developed at the coordinating site (Department of Psychiatry and Psychotherapy, University of Düsseldorf) and has shown efficacy in schizophrenia (23, 25). In the present study, TAR is extended by several behavioral exercises from typical social skills training programs and from the “Playful training of affective resources” program developed at the University of Tübingen (34). These parts of the ISST are not simply concatenated but are tightly integrated to produce additional benefits, i.e., behavioral skills like conversation skills, assertiveness skills, and vocational/work skills are always addressed in conjunction with the respective social cognitive functions (e.g., social and affect perception, ToM); exercises focusing mainly on either receptive and cognitive functions or on expressive and interactional skills are redesigned in such way that they always contain some reference to the other functions and skills. Furthermore, ISST always considers the transfer and implementation of skills into individual problem areas in real life (as identified in the first treatment session). It follows essential treatment characteristics identified in retrospective reviews (18, 35); in particular, it explicitly uses strategy training (beyond drills and practice), personalization, and contextualization to enhance transfer into real life. Thus, ISST strives to integrate social behavioral with social cognitive training principles by both emphasizing explicit practical exercises of learned social cognitive skills (e.g., in role plays and also in real-life exercises in the community) and specifically fostering cognitive comprehension of behavioral skills exercises.

The study compares ISST with an active control intervention, NCRT, which targets impairments in attention, memory, and executive functions. NCRT is based on a neurocognitive training used in our earlier studies (23, 25, 36) but has been enriched with additional exercises from a neuropsychological rehabilitation program (37). NCRT was chosen as the active control treatment because it can be exactly matched to ISST in dose, mode, and application scheme. Thus, using NCRT as the control condition ensures a comparable amount of therapeutic attention and commitment to therapy in both groups, which would not be possible to a similar extent with other control conditions such as treatment as usual or supportive therapy. Moreover, using NCRT ensures that both treatments have a comparable therapeutic focus, i.e. on cognitive impairments. And finally, such an active control condition is more acceptable to patients than treatment as usual without an additional therapy component. However, NCRT not only targets a different subset of cognitive processes but also follows a different kind of treatment strategy: In contrast to ISST, NCRT uses a drill and practice approach, i.e., the treatment strategy preferentially used in neurocognitive remediation programs (38). In former studies, such cognitive drill and practice training without adjunctive psychosocial rehabilitation had no significant impact on social functioning (39). This, NCRT is an appropriate active control treatment.

The study applies both ISST and NCRT as add-ons to treatment as usual, i.e., to pharmacological and/or psychological treatment as indicated by the treating clinical therapist. Furthermore, both therapies are conducted in accordance with detailed treatment manuals. All study therapists are specially trained in applying the therapies, and after each treatment session, they record adherence to the manual by completing protocol forms.

In both conditions, treatment lasts 6 months and comprises a total of 18 sessions (Table 1). It starts with 10 individual sessions (one session per week in week 1–10). The first session serves to identify individual impairments and resources, as well as troubling situations, and the next nine sessions provide basic training of the targeted cognitive processes, i.e., social cognitive processes in ISST and neurocognitive processes in NCRT. Subsequently, these individual sessions are complemented by five group sessions held every 2 weeks in the lab (sessions 11–15, week 12–20) and then two individual sessions in everyday life situations to enhance the transfer of skills (sessions 16–17, week 22–24). In the ISST group, sessions 11 to 17 are used to practice the trained social cognitive and behavioral skills in both role-play situations that foster interactional behavior and in real life. In the NCRT group, these sessions focus on the trained neurocognitive functions and are structured in such a way that interactional behavior is secondary, i.e., as competition-like rather than cooperative tasks; this approach provides the same amount of group interaction and companionship and the same amount of guided community activity as in the ISST group. The final session of each treatment program comprises individual feedback, in which the therapist and patient together analyze whether the individual goals have been achieved.

**Table 1:** Overview of treatment conditions.

	**Integrated social cognitive and behavioral skill therapy**	**Neurocognitive remediation therapy**
General approach	Predominantly strategy based; as far as possible, personalizes and contextualizes learning activities	Predominantly based on repeated practice
Session 1, individual, week 1	Individual analysis of social-cognitive and social behavioral impairments and resources	Individual analysis of neurocognitive impairments and resources
Session 2–10, individual, week 2–10	Multimodal exercises (PC-based, desk work) that integrate recognition and expression of affect (mimic, prosody, gestures), empathy/theory of mind, and situational understanding	Multimodal exercises (PC-based, desk work) that focus on attention, memory, and executive functioning
Session 11–15, open groups, week 12–20	Group exercises to practice the trained social-cognitive functions and expressive behavior, as well as social skills, in role-play situations to foster interactional behavior	Group exercises to practice the trained neurocognitive functions in competition-like games (to minimize interactional behavior)
Session 16–17, individual, week 22–24	Individual practicing of the trained social-cognitive and social behavioral skills in the community to enhance everyday transfer	Individual practicing of the trained cognitive functions in outdoor activities to enhance everyday transfer
Session 18, individual, week 26	Feedback; analysis of achievements on the basis of the initial problem analysis	Feedback; analysis of achievements on the basis of the initial problem analysis

### Endpoints and Data Collection and Management

To ensure comparability of results, all ESPRIT network studies use the frequency of all-cause discontinuation (ACD) across the 12-month study period, i.e., until V12, as the primary endpoint (Table 2). ACD is defined as (1) not keeping appointments for treatment sessions or diagnostic assessments as scheduled for more than 6 weeks; (2) an inability to reach the participant despite extensive efforts by the study team; (3) termination of study participation by the patient or (4) termination by the study staff (e.g., for clinical reasons); (5) non-compliance with prescribed drug treatment for more than 14 consecutive days; and/or (6) relevant worsening of symptoms (PANSS total score ≥ 75 on consecutive visits over more than 14 days).

**Table 2:** Schedule of enrolment, interventions, and assessments.

	**Screening**	**Treatment**						**Follow-up**		
	**Day−7 to−1**	**Day 0**	**Day 28 ±3**	**Day 63 ±7**	**Day 98 ±7**	**Day 140 ±14**	**Day 182 ± 14**	**≈Day 224 ±21**	**≈Day 273 ±21**	**≈Day 365 ±28**
**Variables**	**V0**	**V1**	**V2**	**V3**	**V4**	**V5**	**V6**	**V8**	**V10**	**V12**
Informed consent	X									
Inclusion/exclusion criteria + Mini International Neuropsychiatric Interview, version 6 (M.I.N.I. 6)	X									
Case history, Childhood Trauma Screener	X									
Other therapies	X						X			X
Randomization	X									
**Efficacy: study continuation and treatment adherence**										
All-cause discontinuation		X	X	X	X	X	X	X	X	X
Service Engagement Scale (SES)		X					X			X
Drug Attitude Inventory (DAI)		X					X			X
Patient Questionnaire on Therapy Expectation and Evaluation (PATHEV)		X					X			X
**Efficacy: social functioning/quality of life**										
Social and Occupational Functioning Assessment Scale (SOFAS)		X	X	X	X	X	X	X	X	X
Functional Remission of General Schizophrenia (FROGS)		X					X			X
University of California Performance-based Skills Assessment (UPSA-Brief)		X					X			X
Quality of Life (WHOQOL-Bref)		X					X			X
Efficacy: cognitive functioning										
**Social cognition:** Pictures of Facial Affect (PFA), Movie for the Assessment of Social Cognition (MASC)		X					X			X
**Neurocognition:** Auditory Verbal Learning Test (VLMT), Digit-Symbol Substitution Test (ZST), Digits forward/backward (ZN), Trail-Making Test (TMT-A, -B)		X					X			X
**Efficacy: clinical status**										
Positive and Negative Syndrome Scale (PANSS)	X	(X)			X		X			X
Calgary Depression Scale for Schizophrenia (CDSS)							X			X
Clinical Global Impression (CGI)		X	X	X	X	X	X	X	X	X
Brief Symptom Inventory (BSI)		X					X			X
**Safety**										
Severe adverse events		X	X	X	X	X	X	X	X	X
Side effects		X					X			X

The underlying criteria correspond with the definition of ACD used in earlier studies by other groups and in consensus guidelines (40–42) and ensure comparability of ESPRIT studies with other studies in the field. Because patients in clinical practice usually would not have to leave psychotherapy merely because of either non-adherence to drug prescriptions or temporary symptom worsening unrelated to psychotherapy, patients who meet criterion (5) or (6) in the present study will be formally documented as fulfilling the ACD criteria, but treatment and assessments will be continued with regard to secondary endpoints (see below). In case of relevant symptom worsening or a suicidal crisis, patients will be motivated by study staff to contact their psychiatrist and/or consider inpatient treatment. At the end of the crisis or inpatient treatment, the study treatment will be continued unless the 6-month treatment period is over.

The primary endpoint was chosen for two main reasons: (1) In psychiatric treatment and care (in particular in patients with schizophrenia), discontinuation or non-adherence is a large problem that prevents patients receiving effective treatment. Thus, this outcome is clinically most relevant, and consequently, ACD has become a sort of standard endpoint that has been used in landmark psychiatric treatment trials such as the Clinical Antipsychotics Trials of Intervention Effectiveness [CATIE; (43, 44)] and the European First Episode Schizophrenia Trial [EUFEST; (40)]. (2) ACD was chosen as the common primary endpoint in all clinical studies by the ESPRIT network to allow for cross-referencing and cross-comparisons between studies and for data pooling to enable pooled predictor analyses. Besides these formal reasons, ACD is justified as an outcome also because poor cognitive functioning is the strongest patient-related predictor of impaired ability to manage medications (45). Moreover, patients with more pronounced cognitive impairments show poor adherence behavior (15) and low service engagement (16). Correspondingly, in cognitive remediation programs a close correlation has been reported between the number of received treatment sessions and a positive treatment outcome (46).

To assess changes in processes primarily addressed by cognitive remediation therapy, the study evaluates additional proximal functional, cognitive, and quality of life measures as secondary outcomes. In particular, these outcomes include assessments of improvements in social and occupational functioning [FROGS; (47), SOFAS, (48)]; performance-based skills [UPSA-Brief; (49)]; quality of life [WHOQUOL-Bref; (50)]; neurocognitive performance, which comprises verbal memory [VLMT; (51)] working memory [ZN; (52)], speed of processing [ZST; (52)], [TMT A; (53)], and executive functioning (TMT-B); and social cognitive performance, which comprises facial affect recognition [PFA; (54)] and ToM [MASC; (55)].

Effects on clinical symptoms are assessed with the Positive and Negative Syndrome Scale [PANSS; (56)], Calgary Depression Scale for Schizophrenia [CDSS, (57)], and Brief Symptom Inventory [BSI, (58)]. In accordance with the guidelines proposed by Velligan et al. (41) for dealing with adherence problems, the Service Engagement Scale [SES; (59)], Drug Attitude Scale [DAI-10; (60)], and Patient Questionnaire on Therapy Expectation and Evaluation [PATHEV; (61)] are used to elucidate the reasons for patients dropping out and describe the changes in patients' adherence behavior.

Safety endpoints are (i) death by suicide, (ii) severe suicidal crisis/attempt [score for CDSS Item 8, ≥ 2 (57)], and (iii) severe symptom exacerbation [score for Clinical Global impression Scale, Item 2, ≥ 6 (62)] and are assessed monthly as severe adverse events during the formal assessment sessions. Moreover, the approximately weekly treatment sessions also allow for careful informal monitoring of such safety variables.

At each study site, data are entered into a web-based data management system (TrialMaster^TM^, Anjusoftware.com) by means of an electronic data capture (electronic case report form). The IT infrastructure and data management are provided by the Clinical Trials Center Cologne (CTCC, S. Schmied), an independent academic research organization. To ensure the completeness and quality of the data, CTCC also provided regular on-site data monitoring, supported by a monitor from the coordinating site.

The safety, integrity, and progress of the study is regularly reviewed by an independent committee of five international experts in psychiatry and medical statistics (Data Safety Monitoring Committee).

### Power Calculation

On the basis of data from two larger German and European therapy studies (17, 40), we assumed a one-year discontinuation rate of 68% in the control group. A reduction of 20% in the intervention group was considered as clinically relevant and was shown by Galderisi, Piegari (63) to be realistic. To verify a difference of 20% in the 1-year discontinuation rate between the intervention and control groups by two-sided testing with an error probability of α = 0.05 and a power of 1 – β = 0.8 in the chi-square test, *n* = 90 participants have to be included in each study arm (calculated with the software Power and Sample Size Calculation [PS] 3.0.43, freely available at https://biostat.mc.vanderbilt.edu/PowerSampleSize). When time-to-event is considered, the power of the corresponding Wald test from Cox regression is probably higher. Because the overall dropout rate is used as the primary outcome variable, there is no need to compensate for dropouts by including additional study participants. Furthermore, using the overall dropout rate as the primary outcome variable will ensure that the number of missing values for this measure is (close to) zero.

For the analysis of secondary endpoints, the sample size will allow an effect size of 0.42 (80% power, two-sided testing) to be detected, which is the value obtained for functional outcome in similar studies, albeit in very small samples (33, 64).

### Statistical Analysis

To avoid any bias in the data analyses, data will be analyzed primarily according to the intention-to-treat (ITT) principle. The per-protocol set will be analyzed secondarily. Statistical analyses will be performed by an independent statistician at IMSB (M. Hellmich) who will have no information on treatment allocation.

The superiority of ISST over NCRT regarding the primary endpoint ACD over 1 year will be tested by a Cox regression, stratified by study site, with the main effects intervention (ISST/NCRT), antipsychotic dose at baseline (standardized across patients as chlorpromazine equivalents), age, and sex, without interaction (Wald test at two-sided α = 5%). Adjusted hazard ratios with 95% confidence intervals will be calculated.

Mixed models for repeated measures (MMRM) will be used to describe and evaluate changes in secondary efficacy variables (e.g., regarding social and occupational functioning, quality of life, neurocognition, social cognition, and clinical symptoms) and safety variables over time with the fixed effects intervention, time, and intervention^*^time, baseline, and study site (random effects patterns; heterogenous first-order autoregressive [ARH (1)] structured variance-covariance matrix). The pattern of missing values (missing completely at random/MCAR vs. missing at random/MAR vs. not missing at random/NMAR) will be investigated, and the impact of various strategies for handling missing values be explored in a sensitivity analysis. Serious adverse events will be summarized by type of event, severity, and relatedness to study treatment. Subgroup analyses will be performed by sex and study site.

### Trial Status

The ESPRIT ISST Trial has completed recruitment and the follow-up assessments. Data clearing is still underway and must be completed before final statistical analyses can be performed. First results are expected in autumn 2022.

## Discussion

Persisting impairments in psychosocial functioning are a defining and debilitating feature of schizophrenia that preclude patients from living a satisfying life and thus hamper full recovery (1, 2). Essential factors limiting psychosocial functioning are impairments in neurocognition, social cognition and social behavioral skills. Traditional drug treatments and psychosocial therapy, and the available specific treatment strategies that directly target such key determinants of functional outcome have produced only moderate effects, leaving an urgent need for further optimization of treatment. In particular, cognitive remediation has shown general efficacy in improving cognition and social functioning, but effects are still comparatively small and heterogeneous. This heterogeneity also prevents cognitive remediation therapies from being consistently recommended by international guidelines: Although several scientific societies explicitly recommend cognitive remediation (65–68) others refrain from doing so (69, 70), mostly because of the scarce evidence for long-term effects in particular on social outcome, the heterogeneity of remediation programs, and the relatively small sample sizes studied. Thus, methodologically rigorous, appropriately powered RCTs that use longer follow-up periods and adhere to recently identified essential treatment elements, as used in the present ISST trial, are urgently needed.

Moderator analyses of recent reviews and meta-analyses (18, 19, 31) suggest that combined cognitive remediation and social behavioral training is the most promising approach to obtain better clinical effects on psychosocial functioning and quality of life. However, there is only scarce direct experimental evidence for a superiority of combined treatment (33). Moreover, this former study used neurocognitive remediation as the cognitive component of the treatment program and added behavioral-based training of social competence at the end of cognitive remediation. Because there is evidence of a superior effect of social cognitive remediation—as compared with neurocognitive remediation—on social outcome in schizophrenia, the present study integrates, rather than concatenates, social cognitive and behavioral skill therapy to obtain synergistic effects. Moreover, ISST uses a strategy-based treatment approach that has been proven to enhance the transfer of the trained skills into patients' daily lives (71). The effects on social outcome of this integrated social cognitive and social behavioral skills training are being compared with the effects of a more traditional drill and practice-based neurocognitive training. Consequently, the trial will help to identify the optimal treatment approach for impaired social functioning, a deficit self-rated by patients as their area of greatest unmet need (2, 5). Enhancing prevention and recovery in patients with schizophrenia through innovative treatments also is the general aim of the German ESPRIT research network, which is conducting the present study.

Our study design has several strengths. The study will include a comparatively large sample of 2^*^90 patients and thus be sufficiently powered to detect the expected moderate treatment effect, and it will follow patients for 6 months after completion of treatment to evaluate whether possible effects persist. Furthermore, the study uses strict methodology within a multicenter design; i.e., patients are randomly allocated to treatment groups independent of therapists and investigators; assessments are performed by independent raters who are blind to treatment allocation, and the success of blinding is controlled in each assessment session; treatment conditions follow detailed manuals, and adherence to manuals is controlled by treatment session protocols; data management and statistical analyses will be conducted independently from the trial research team by the CTCC (data management) and IMSB (statistics) at the University of Cologne; and statistical analyses use mainly an ITT approach. Moreover, monitoring of severe adverse events as safety indicators is conducted throughout the therapy and follow-up periods to obtain detailed information on the safety of cognitive remediation therapy.

On the one hand, the use of an active control treatment, which almost perfectly controls for unspecific treatment effects, could be viewed as a further strength of the study. However, on the other hand, the active control condition may also be a weakness if specific treatment effects are smaller than expected and thus do not lead to detectable differences between treatment conditions. Evidence indicates that differences in treatment effects will be large enough to enable them to be detected, given that social cognitive remediation and social behavioral training have shown beneficial effects on functional outcome (22, 29) whereas drill and practice-based neurocognitive remediation has shown only marginal effects (39). If the expected differences are not found between treatment conditions, inclusion of a third treatment condition containing treatment as usual without any cognitive remediation and social skills training could increase interpretability of the results, i.e., whether both treatments are equally effective or whether there is no treatment effect at all. However, both pragmatic and ethical reasons precluded the use of such a three-group approach because the availability of patients is limited and depriving patients of treatment components such as cognitive and social skills training, which is already established in many hospitals, seemed inappropriate.

In conclusion, upon completion this study will be one of the largest and methodologically most rigorous clinical trials on the efficacy of cognitive remediation approaches in schizophrenia. Not only will it help to identify the optimal treatment for improving psychosocial functioning and thus recovery in patients with schizophrenia, but it will also allow us to draw conclusions about the factors the influence and mediate the effects of cognitive remediation in these patients.

## ISST-Study Group

The study is guaranteed by the ISST study group, which consists of the following people: W. Wölwer, S. Abresch, N. Frommann, A. Lowe, D. Kamp, P. Ockenfelds, K. Weide, F. Pessanha, S. Dinse (Düsseldorf); A. Philipsen, R. Hurlemann, J. Schultz, N. Striepens, U. Darrelmann, C. Kloss, S. Wasserthal, H. Högenauer, N. Schumacher (Bonn); F. Jessen, J. Kambeitz, C. Baldermann, A. Muthesius, C. Doll, H. Schneegans, A. Ferrari, G. Kolb, T. Haidl, D. Zeus, T. Pilgram, M. Rohde, P. Albert-Porcar, S. Hölzer, M. Hellmich, K. Kuhr, K. Rosenberger, D. Kraus, S. Schmied, U. Bergmann, F. Scheckenbach, A. Montada (Cologne); S. Klingberg, D. Wildgruber, U. Hermanutz, J. Richter, J. Vonderschmitt, L. Hölz (Tübingen); A. Bechdolf, K. Leopold, S. Siebert, F. Seidel, E.S. Blanke (Berlin), A. Brockhaus-Dumke, X. Solojenkina, B. Klos, E. Rosenbauer, S. Cinar, L. Herdt, F. Henrich, S. Neff (Alzey), A. Meyer-Lindenberg (Mannheim)

## Ethics Statement

The studies involving human participants were reviewed and approved by Ethics Committee of the Medical Faculty, University of Düsseldorf (reference number: 5333R). The local Ethics Committees of the other participating centers have acknowledged this vote. The patients/participants provided their written informed consent to participate in this study.

## Author Contributions

WW is the principal investigator and grant holder of the ISST study. WW, NF, AL, DK, and KW designed the study. WW, AB, AB-D, RH, AM, and SK are the local principal investigators. MH is the statistician of the study. SS provided remote data entry and data management. AM-L is the representative of the study sponsor. All authors read and approved the final manuscript.

## Funding

This study is publicly funded by the German Federal Ministry of Education and Research (Bundesministerium für Bildung und Forschung, BMBF), grant number 01EE1407F. The study is part of the BMBF-funded research network ESPRIT (Enhancing Schizophrenia Prevention and Recovery through Innovative Treatments, Coordinator: A. Meyer-Lindenberg, Mannheim). The funding agency selected projects on the basis of the vote of an international review board; it does not exert any influence on the concept or execution of the trial.

## Conflict of Interest

The authors declare that the research was conducted in the absence of any commercial or financial relationships that could be construed as a potential conflict of interest.

## Publisher's Note

All claims expressed in this article are solely those of the authors and do not necessarily represent those of their affiliated organizations, or those of the publisher, the editors and the reviewers. Any product that may be evaluated in this article, or claim that may be made by its manufacturer, is not guaranteed or endorsed by the publisher.

## Abbreviations

CDSS: Calgary Depression Rating Scale for Schizophrenia
CTCC: Clinical Trials Center Cologne
FROGS: Functional Remission of General Schizophrenia
IMSB: Institute of Medical Statistics and Computational Biology
MASC: Movie for the Assessment of Social Cognition
PANSS: Positive and Negative Syndrome Scale
PFA: Pictures of Facial Affect
RCT: Randomized Controlled Trial
SOFAS: Social and Occupational Functioning Assessment Scale
TMT-A/-B: Trail-Making Test Version A/B
UPSA-Brief: University of California Performance-based Skills Assessment
VLMT: Auditory Verbal Learning Test
WHOQOL-Bref: World Health Organization Quality of Life
ZN: Digits forward/backward
ZST: Digit-Symbol Substitution.
